# When home is a dangerous place: the cost of falls among older people living in the community

**DOI:** 10.1371/journal.pone.0353755

**Published:** 2026-08-05

**Authors:** Javiera Cartagena-Farías, José-Luis Fernández, Wagner Silva-Ribeiro, Nicola Brimblecombe

**Affiliations:** Care Policy and Evaluation Centre (CPEC), London School of Economics and Political Science, London, United Kingdom; Pusan National University College of Economics and International Trade, KOREA, REPUBLIC OF

## Abstract

Falls among older people are common, and they may have consequences such as fractures and injuries. Falls may also reduce older people’s independence and wellbeing and increase demand for both healthcare and social care services. This research aims to investigate whether older people living in the community who experience poor housing conditions face a greater risk of falls than those living in better-quality housing after controlling for other risk factors. In particular, we aimed to (i) identify housing-related drivers of falls, and (ii) estimate associated costs. Using data from the English Longitudinal Study of Ageing (ELSA), covering the period from 2002/03–2021/23, we performed a combination of cross-lag and Heckman selection models to address endogeneity and selection bias. We also developed a decision tree model to estimate associated healthcare and social care costs. We found that those living in homes with electrical or plumbing problems, as well as those lacking space, are more likely to experience a fall. Similarly, we found that older people living in homes that are ‘too dark’ are more likely to experience a fall and to visit a healthcare service as a consequence. We estimated that the annual cost of falls attributable to poor housing conditions is £1.2 billion. Poor housing conditions significantly increase the risk of falls among older people. Associated costs could be avoided. Viewing housing improvements as part of an integrated, neighbourhood-level falls prevention strategy could yield significant benefits for individuals and for the sustainability of health and care systems.

## Introduction

In England, one in three older people living in their own home experience at least one fall a year [1]. Falls often result in physical injuries, but they may also reduce older people’s confidence, independence, and wellbeing, and thus increase demand for both healthcare and social care services [2–4]. The need for preventative strategies has been widely recognised, and as such, guidelines to minimise or avoid their prevalence have been developed [5–6]

Much of the literature exploring the factors associated with an increased risk of falls has emphasised the identification of individual-level characteristics, including age, gender, ethnicity, frailty status, balance problems, vision impairment, and long-term conditions such as cognitive decline [7–9]. For those at risk, clinical and behavioural interventions have been the norm. For example, vitamin D supplementation programmes have been implemented to address deficiencies associated with bone health, alongside exercise classes designed to improve balance and muscle strength [10–13].

More recently, the role of the home environment has gained increasing attention as a potential preventative strategy to avoid or reduce the risk of falls. In this regard, home adaptations (such as grab rails, ramps, and improved access), assistive technologies, and innovations have demonstrated relative effectiveness [14–15]. Yet, the role played by broader housing conditions (e.g., cold indoor temperatures and lack of space) remains less well explored. These factors may present significant risks for older adults, particularly those living in their own homes, where most falls occur [16].

Given this research gap, this study aimed to explore whether older people (aged 65 years and over) living in the community who experience poor housing conditions (after controlling for other risk factors) face a greater risk of falls than those living in better-quality housing. More specifically, we aimed to (i) identify housing problems that increase the risk of falls, and (ii) estimate the associated healthcare and formal care service costs. By doing so, this study contributes to a more comprehensive understanding of environmental (and potentially avoidable) determinants of falls and their consequences for care utilisation.

### Poor housing conditions and the risk of falls: Previous evidence

Previous evidence has highlighted the importance of the home environment for falls prevention, though with varying emphases. Systematic reviews have demonstrated that home safety interventions, often delivered as part of multifactorial programmes, can reduce the prevalence of falls among older adults living in the community [17–19]. These interventions usually involve relevant professionals assessing home environments and identifying hazards, and then recommending modifications such as fixing loose carpets, repairing poor stair conditions, dealing with slippery floors, improving lighting, and/or clearing cluttered pathways. While these approaches seem promising, they typically adopt a holistic model in which multiple risk factors are addressed simultaneously, making it difficult to isolate the specific role of housing quality in falls prevention.

Evidence linking poor housing conditions directly to falls is more limited. International and national studies have increasingly recognised the importance of housing quality [20]. Lack of space, for instance, may increase tripping hazards, obstruct visibility, and movement [21], while low indoor temperatures have been associated with poorer physical functioning and a greater risk of accidents [22]. Policy initiatives, such as the “Boiler on Prescription” trial in Sunderland, and other areas suggest that improving indoor temperatures can reduce falls and hospital admissions [23], although robust evaluations remain scarce.

Despite these attempts, most of the existing evidence has prioritised housing adaptations over housing quality. Adaptation-focused interventions have been demonstrated to have preventative potential, but they may not address the more systemic risks arising from substandard housing conditions. Moreover, much of the available economic evidence on fall costs is confined to hospital or residential settings, with limited attention to community-dwelling. In Scotland, community-based falls in older adults were estimated to cost over £470 million in healthcare and social care expenditure [24], underscoring the urgency of preventative strategies that extend beyond clinical and behavioural domains. Overall, while environmental interventions are recognised as an important component of falls prevention, research evidence remains fragmented and underdeveloped.

## Data

This study uses a representative national sample from the English Longitudinal Study of Ageing (ELSA), waves 1–10, covering the years 2002−2003 (wave 1) to 2021−2023 (wave 10) [25]. Wave 10 covered the period from 2021 to 2023, reflecting delays and disruptions to primary data collection associated with the COVID-19 pandemic. ELSA includes individuals aged 50 years and older and their partners, but we restricted the sample to those aged 65 and over to maintain widely used definitions for older people. Approximately 5,500 individuals were included in each wave. Information was also available on socio-demographic characteristics, care needs, health status, and housing-related variables.

An ethics application was submitted through the London School of Economics and Political Science Research Ethics process (Ref: 332823). Following review, it was concluded that formal ethics approval was not required for this study because the project used only secondary data sources. For further information, please contact research.ethics@lse.ac.uk.

### Falls

In ELSA, respondents were asked whether they had fallen since their last interview: *“Have you fallen down in the last two years (for any reason)?”* This was coded as a binary variable (0 = No, 1 = Yes). Those who reported a fall were further asked: *“In any of these falls, did you injure yourself seriously enough to need medical treatment?”* This was also coded as a binary variable (1 = Yes, 0 = No). Information on the specific type of medical service was not available.

### Housing

ELSA collects detailed information on housing characteristics and quality. These include the number of household members, number of rooms, and whether adaptations had been installed (e.g., handrails, automatic doors, and alerting devices). Self-reported housing problems were also recorded, including issues with lack of space, general disrepair, cold indoor temperatures, lack of light, noise, condensation, water leaks from roofs or windows, electrical or plumbing faults, infestations, and rising damp. Each problem was coded as 1 if present and 0 if absent. We also included fuel poverty as a potential factor increasing risk of falls. Here (due to data limitation on energy consumption and housing insulation levels), we defined fuel poverty as those households that spend 10% or more of their income on energy (gas and electricity) bills. The latter was coded 0 if individuals are not living in fuel poverty and 1 if they are.

### Socio-demographic characteristics, care needs, and health status

Socio-demographic information included gender (0 = male, 1 = female), marital status (0 = single, 1 = married), long-standing illness (0 = no, 1 = yes), living alone (0 = no, 1 = yes), age, and self-rated health (0 = fair/poor/very poor, 1 = good/very good). Care needs are also found as the need for support with Activities of Daily Living (ADLs) and Instrumental Activities of Daily Living (IADLs). ADLs include support with going to the toilet, eating, bathing, personal care, etc. and IADLs include the need for support with tasks such as shopping, making calls, taking medication, and managing money, among other things. Once again, the need for support was coded with a 1, and 0 otherwise.

### Cost of healthcare and social care services

We obtained the cost of formal care and healthcare services using PSSRU and NHS unit costs for the year 2023/2024 [26–27]. Cost of NHS 111 services were not available, but an estimation was used based on [28]. Older people (65+) population size for England was obtained from Census 2021 [29].

## Methodology

Investigating the causal association between poor housing conditions and the risk of falls among older adults presents two key methodological challenges. The first is reverse causality: while housing conditions may affect the probability of experiencing a fall, falls themselves may also increase the likelihood of housing disrepair. For example, a fall may reduce mobility, thereby limiting an individual’s ability to carry out repairs or maintain their home. The second challenge is selection bias, as housing problems may be associated with higher mortality between survey waves. If those living in poor quality housing are more likely to die or move to residential care before subsequent interviews, the observed sample may underestimate the true effect of housing on the risk of falls. To address these challenges, we apply two complementary approaches: a cross-lagged panel model and a Heckman selection model.

### Cross-lagged panel model

To tackle reverse causality, we estimate the effect of housing conditions measured at time *t–1* on the risk of falls at time *t*. This is a standard, simple and effective way to rule out the possibility of a bidirectional relationship as the past can influence the future, but not the other way around [30]. The model controls for socio-demographic characteristics, long-standing illness, and history of previous falls at *t–1*. The main specification is given by:


$$\begin{array}{c} P\left({Fall}_{it}\right)=\;\alpha +\;\gamma X_{i,t-\text{1}}+{\beta HousingConditions}_{i,t-\text{1}}+\;\epsilon_{it} \end{array}$$ (1)

Where $P\left({Fall}_{it}\right)$ is a binary indicator of whether individual i experienced a fall at time t, $X_{i,t-\text{1}}$ represents socio-demographic, health-related factors, previous falls, and local deprivation levels (quintiles of the Index of Multiple Deprivation). ${HousingConditions}_{i,t-\text{1}}$ captures reported housing quality issues as binary variables), and $\epsilon_{it}$ represents the error term. We have performed, in the first instance, a panel-data (Random Effects) probit regression model, with clustered and robust standard errors.

### Heckman selection model

To account for potential bias arising from an association between poor housing conditions and mortality or attrition between waves, we estimated a two-step Heckman correction model [31]. The first-stage selection equation predicts the probability of an individual remaining in the sample at time *t*, using auxiliary variables correlated with sample retention but not directly with falls outcomes. We tested several instruments, including smoking status, number of completed survey waves, incidence of heart attack, exposure to pollution, enjoyment of social company, receipt of bequest (£50k), receipt of attendance allowance, and combinations thereof. The final specification employed smoking status (0 = no, 1 = yes) and number of survey waves participated in (ranging from 1 to 10), which satisfied relevance and exclusion criteria.

The outcome (second stage) equation is then estimated as:


$$\begin{array}{c} P\left({Fall}_{it}\right)=\alpha +{\gamma X}_{i,t-\text{1}}+\beta {HousingConditions}_{i,t-\text{1}}+\delta \lambda_{it}+\varepsilon_{it} \end{array}$$ (2)

Where $\lambda_{it}$ is the inverse Mills ratio (IMR) derived from the first-stage selection equation

This modelling framework allows us to (i) reduce concerns of reverse causality, by using lagged housing measures, and (ii) correct for potential sample selection bias. Together, these approaches provide a more robust estimate of the causal impact of poor housing quality on falls among older adults in England. To aid interpretation, we report marginal effects derived from probit models for housing conditions. Unlike raw probit coefficients, which represent changes in the unobserved latent index, marginal effects express the change in the probability of experiencing a fall associated with a one-unit change in the explanatory variable. These are calculated as the product of the estimated coefficient and the standard normal density evaluated at the mean of covariates. Stata 18 [32] was used to perform all analyses.

### Decision tree model

**Fig 1:**
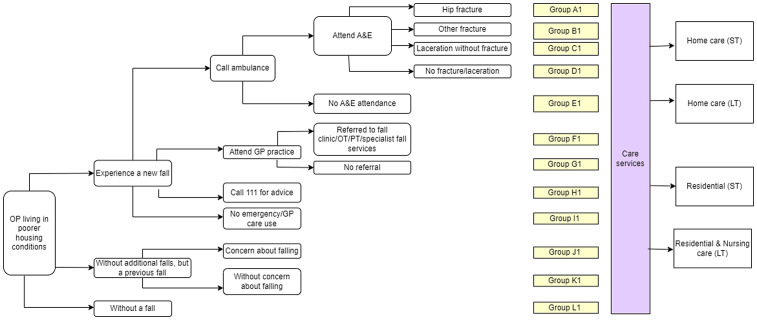
Decision tree pathways.

We developed a decision-tree model to estimate the healthcare and (formal) care consequences and costs of falls among older people (65+) living in poor housing conditions (defined here as lack of space, poor lighting, and plumbing/electrical issues). The model follows a cohort of older adults through mutually exclusive care pathways triggered by a new fall (e.g., calling an ambulance and attending A&E, GP attendance with or without referral, no healthcare use), which assigns probabilities to transitions along the tree and attaches unit costs to each service use. For each terminal node, we also included the subsequent likelihood of short-term and long-term community or residential care. Total annual costs were obtained by aggregating costs across pathways, scaled up to the population level using ONS population estimates for England taken from Census 2021 [29]. Fig 1 presents a visual representation of the developed decision tree:

### Developing pathways and assumptions

The decision model was mostly designed in a deterministic manner. In order to estimate the additional cost associated to falls caused by poor quality housing, we considered all older people in the sample, and we estimated the percentage change (i.e., the additional effect) in the risk of falls associated with living in poor housing conditions. To do this, we applied a multinomial probit Heckman selection model (the only non-deterministic part of the model) with three main outcomes: (i) having a fall at time *t*; (ii) not having a fall at *t* but having had a previous fall at *t-1*; and (iii) not having experienced a fall. The model took the same form as in Equation 2 above.

The estimated coefficient β was converted into a marginal effect, allowing interpretation as the percentage change in the probability of falls associated with living in poor housing, with good housing as the reference category.

Probability for downstream events in the tree (e.g., calling an ambulance, of attending A&E, of sustaining a hip fracture, referral to falls services or OT/PT) were drawn from the published literature and policy documents (see Table 1). Unit costs for ambulance calls, A&E attendance, hospital treatment (hip fracture, other fracture, laceration), home care and institutional care were taken from established UK sources and are presented in Table 2. Some assumptions were also required due to the lack of available data.

**Table 1:** Parameters and assumptions in decision tree model.

Outcome		Pathways	%	Source
	1	OP living in poor housing conditions	26.50%	ELSA
	1.1	OP with a new fall (living in poor housing conditions)	6.20%	ELSA (regression model)
	1.1.1	OP calls ambulance	12.00%	Cockayne, et al. (2021) [16]
	1.1.1.1	OP attend A&E after a fall and after ambulance	87.00%	Calculation: 100-x%
A1	1.1.1.1.1	OP with hip fracture attending A&E (after a fall and ambulance)	6.00%	Craig, et al. (2013) [24]
		Prob. Institutional Care (permanent)	8.10%	Hawley, et al. (2022) [34]
		Prob. Institutional Care (intermediate)	19.00%	Hawley, et al. (2022) [34]
		Prob. Home Care (permanent)	20.20%	Calculation: 100-x%
		Prob. Home Care (short term)	24.70%	Png et al., 2020 [35]
		Prob. No Home Care/Institutional care	28.00%	Patel, et al. (2022) [36]
B1	1.1.1.1.2	OP with other fracture attending A&E (after a fall and ambulance)	12.50%	Pighills, et al. (2021) [37]
		Prob. Institutional Care (permanent)	8.10%	Hawley, et al. (2022) [34]
		Prob. Institutional Care (intermediate)	19.00%	Hawley, et al. (2022) [34]
		Prob. Home Care (permanent)	20.20%	Calculation: 100-x%
		Prob. Home Care (short term)	24.70%	Png et al. (2020) [35]
		Prob. No Home Care/Institutional care	28.00%	Patel, et al. (2022) [36]
C1	1.1.1.1.3	OP with laceration attending A&E (after a fall and ambulance)	43.00%	Cockayne, et al. (2021) [16]
		Prob. Institutional Care (permanent)	1.62%	Assumption (20% of fracture)
		Prob. Institutional Care (intermediate)	3.80%	Assumption (20% of fracture)
		Prob. Home Care (permanent)	4.04%	Assumption (20% of fracture)
		Prob. Home Care (short term)	4.94%	Assumption (20% of fracture)
		Prob. No Home Care/Institutional care	85.60%	Assumption (20% of fracture)
D1	1.1.1.1.4	OP without fracture/laceration attending A&E (after a fall and ambulance)	38.50%	Calculation: 100-x%
		Prob. Institutional Care (permanent)	0.32%	Assumption (20% of laceration)
		Prob. Institutional Care (intermediate)	0.76%	Assumption (20% of laceration)
		Prob. Home Care (permanent)	0.81%	Assumption (20% of laceration)
		Prob. Home Care (short term)	0.99%	Assumption (20% of laceration)
		Prob. No Home Care/Institutional care	98.87%	Assumption (20% of laceration)
E1	1.1.1.2	OP don’t attend A&E after ambulance	13.00%	Mason, et al. (2007) [38]
		Prob. Institutional Care (permanent)	0.06%	Assumption (20% of without/injury)
		Prob. Institutional Care (intermediate)	0.15%	Assumption (20% of without/injury)
		Prob. Home Care (permanent)	0.16%	Assumption (20% of without/injury)
		Prob. Home Care (short term)	0.20%	Assumption (20% of without/injury)
		Prob. No Home Care/Institutional care	99.77%	Assumption (20% of without/injury)
	1.1.2	OP attend GP (after a fall)	10.00%	Craig et al. (2013) [24]
F1	1.1.2.1	OP Referred to Fall clinic/OT after contacting GP (after a fall)	81.10%	Mackenzie & McIntyre (2019) [39]
		Prob. Institutional Care (permanent)	0.01%	Assumption (20% No A&E attendance)
		Prob. Institutional Care (intermediate)	0.03%	Assumption (20% No A&E attendance)
		Prob. Home Care (permanent)	0.81%	Assumption (20% No A&E attendance)
		Prob. Home Care (short term)	0.99%	Assumption (20% No A&E attendance)
		Prob. No Home Care/Institutional care	99.18%	Assumption (20% No A&E attendance)
G1	1.1.2.2	No referral after contacting GP (after a fall)	18.90%	Calculation: 100-x%
		Prob. Institutional Care (permanent)	0.01%	Assumption (20% No A&E attendance)
		Prob. Institutional Care (intermediate)	0.03%	Assumption (20% No A&E attendance)
		Prob. Home Care (permanent)	0.81%	Assumption (20% No A&E attendance)
		Prob. Home Care (short term)	0.99%	Assumption (20% No A&E attendance)
		Prob. No Home Care/Institutional care	99.18%	Assumption (20% No A&E attendance)
H1	1.1.3	Call 111 for advice	30.00%	Calculation: 100-x%
		Prob. Institutional Care (permanent)	0.00%	Assumption (20% with GP attendance)
		Prob. Institutional Care (intermediate)	0.01%	Assumption (20% with GP attendance)
		Prob. Home Care (permanent)	0.16%	Assumption (20% with GP attendance)
		Prob. Home Care (short term)	0.20%	Assumption (20% with GP attendance)
		Prob. No Home Care/Institutional care	99.84%	Assumption (20% with GP attendance)
I1	1.1.4	OP doesn’t attend GP/call emergency services (after a fall)	48.00%	Cockayne, et al. (2021) [16]
		Prob. Institutional Care (permanent)	0.00%	Assumption (20% with Calling 111)
		Prob. Institutional Care (intermediate)	0.00%	Assumption (20% with Calling 111)
		Prob. Home Care (permanent)	0.03%	Assumption (20% with Calling 111)
		Prob. Home Care (short term)	0.04%	Assumption (20% with Calling 111)
		Prob. No Home Care/Institutional care	99.97%	Assumption (20% with Calling 111)
	1.2	OP without a new fall (but previous fall)	2.00%	Pighills, et al. (2021) [37]
J1	1.2.1	OP with fear of falling (no fall)	42.00%	Lavedán et al. (2018) [40]
		Prob. Institutional Care (permanent)	0.00%	Assumption (20% with New fall, no healthcare use)
		Prob. Institutional Care (intermediate)	0.00%	Assumption (20% with New fall, no healthcare use)
		Prob. Home Care (permanent)	0.00%	Assumption (20% with New fall, no healthcare use)
		Prob. Home Care (short term)	0.00%	Assumption (20% with New fall, no healthcare use)
		Prob. No Home Care/Institutional care	100.00%	Assumption (20% with New fall, no healthcare use)
K1	1.2.2	OP without fear of falling (no fall)	58.00%	Calculation: 100-x%
		Prob. Institutional Care (permanent)	0.00%	Assumption
		Prob. Institutional Care (intermediate)	0.00%	Assumption
		Prob. Home Care (permanent)	0.00%	Assumption
		Prob. Home Care (short term)	0.00%	Assumption
		Prob. No Home Care/Institutional care	100.00%	Assumption
L1	1.3	OP without a fall	92.00%	Calculation: 100-x%
		Prob. Institutional Care (permanent)	0.00%	Assumption
		Prob. Institutional Care (intermediate)	0.00%	Assumption
		Prob. Home Care (permanent)	0.00%	Assumption
		Prob. Home Care (short term)	0.00%	Assumption
		Prob. No Home Care/Institutional care	100.00%	Assumption
Measurement	Frequency	Services	LoS	Source
Median number of weeks	LT	Residential care	Survival Curve estimation	Forder and Fernandez (2011) [33]
Median number of weeks	ST	Residential care	6	Age UK (2025) [41]
Median number of weeks	LT	Home care	17	Beresford et al. (2019) [42]
Median number of weeks	ST	Home care	6	NHS (2025) [43]
Median number of weeks	1 visit per week	OT falls prevention programme	12	Age UK (2024) [44]
Population				Source
OP (65+) living in England (N)			56,490,047	Census (2021) [29]

**Table 2:** Unit costs.

Service	Unit Cost (£)	Source
Cost of ambulance call (after a fall and hip fracture)	£250	NHS England 2023/24 (National cost collection) [27]
Cost AE attendance (Emergency Care)	£273	NHS England 2023/24 (National cost collection) [27]
Cost Hip Fracture (hospital)	£12,143	NHS England 2023/24 (National cost collection) [27]
Cost of Fracture (hospital)	£6,215	NHS England 2023/24 (National cost collection) [27]
Cost of laceration/injury	£2,028	NHS England 2023/24 (National cost collection) [27]
Cost GP visit (with referral to OT after a new fall)	£117	PSSRU Unit Costs of Health and Social Care 2023/24; Jones et al. (2024) [26]
Cost Referral to OT/PT	£54	PSSRU Unit Costs of Health and Social Care 2023/24; Jones et al. (2024) [26]
Cost calling 111	£107	Turner et al., 2021 (inc. inflation) [28]
Cost residential care (week)	£1,036	PSSRU Unit Costs of Health and Social Care 2023/24; Jones et al. (2024) [26]
Cost nursing care (week)	£1,382	PSSRU Unit Costs of Health and Social Care 2023/24; Jones et al. (2024) [26]
Cost Home care (month)	£403	PSSRU Unit Costs of Health and Social Care 2023/24; Jones et al. (2024) [26]

The decision tree begins with the subpopulation of older people living in poor housing and branches into: those who experience a new fall in the year and those who do not. For fallers, the tree routes individuals through possible immediate responses (call ambulance, A&E attendance, and categorised clinical outcomes; call 111; attend GP; no healthcare use), then into consequent care states (no further care, short-term home care, long-term home care, short-term institutional care, long-term institutional care). For each branch, the estimated cost is computed as the product of the branch probability and summed unit costs along that branch. Formally, the expected annual cost for branch *j* is:


$$\begin{array}{c} {Cost}_{j}=Prob\left({fall}^{{branch}_{j}}\right)*\sum_{k\epsilon j}{UnitCost}_{k} \end{array}$$ (3)

and


$$\begin{array}{c} Total\;cost=\sum_{j}{Cost}_{j} \end{array}$$ (4)

Total costs were divided into two categories, healthcare and social care related’, and are presented in 2023 prices. Estimations of the latter include local authority-funded and self-funded services.

It is also worth noting that the decision tree model was not populated based on a systematic review. We did ensure, however, that the papers used were relevant (e.g., based on UK data as much as possible) and based on representative samples (as well as other PRISMA-relevant indicators).

### Triangulation

To triangulate our cost estimations, we compared results with published estimates of healthcare and formal care costs associated with falls. In particular, we used Craig et al. (2013) [24] which provides aggregate health and social care cost estimates for older adults experiencing falls in Scotland. To make their findings comparable, we adjusted Craig et al.’s figures for inflation and scaled them by population size (to reflect England rather than Scotland, and to account for differences in the 65 + population). We also took account of the fact that their figures relate to all falls in older people, rather than those specifically attributable to poor housing.

One of the main methodological differences from Craig’s work (in addition to those already mentioned above) is that we estimated the cost of institutional care based on the distribution of length of stay reported by Forder and Fernandez (2011) [33], whereas Craig simplified this by applying the median length of stay. We also included additional healthcare services such as NHS 111 or referrals to an Occupational Therapist by a GP, services that were not considered by previous works. On the other hand, due to the availability of administrative data, they were able to estimate re-admissions, something which we could not consider (although this may be a minor issue given the time cap).

## Results

### Falls prevalence

**Fig 2:**
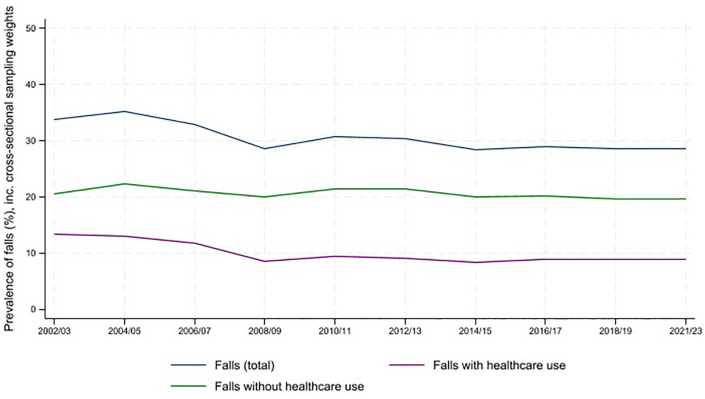
Prevalence of falls among older people living in the community (ELSA waves 1-10).

We started the analysis by estimating the prevalence of falls among older people living in the community in England using the English Longitudinal Study of Ageing – ELSA (using waves 1–10, covering the period 2002–2023). Approximately a third of older individuals (65 years or older) experience one or more falls a year. One in ten older people reported visiting healthcare services due to a fall. These results are consistent with estimations provided by the Office for Health Improvement & Disparities [1], see Fig 2 for more details.

### Poor housing conditions associated to an increased risk of fall

Using a cross-time-lag approach within a Heckman selection model, our results show that those living in homes that have electrical or plumbing issues (β = 0.274; p-value = 0.011) as well as those with lack of space (=0.084; p-value = 0.056) are more likely to experience a fall. Similarly, we found that older people living in homes which are ‘too dark or without enough light’ (β = 0.236; p-value = 0.011) and again have ‘electrical wiring or plumbing issues’ (β = 0.197; p-value = 0.100), are more likely to experience a fall and visit a healthcare service as a consequence, which may be interpreted as a more serious fall. In this analysis we have controlled for: previous fall, age, gender, marital status, education, long-standing illness, wheelchair use, health status, number of ADLs/IADLs, receiving informal care, and tenure.

Due to the collinearity between tenure (privately renting, social housing, and owner occupied) and housing condition, this variable was excluded as sensitivity analysis (column 7 and column 10 in Table 3). As expected, we see an increase in the effect of housing conditions when tenure is removed from the model of falls (β = 0.278; p-value = 0.010 for electrical/plumbing issues and β = 0.096; p-value = 0.031 for lack of space) and to the model of falls with healthcare use (β = 0.197; p-value = 0.100 for electrical/plumbing issues and β = 0.251; p-value = 0.006 for lack of space). Heckman selection model selection bias test indicated that there is no evidence of selection bias (Prob > chi2 = 0.5830). Therefore, we also reported cross-lag only models, for which we obtained very similar results. Other housing-quality issues, such as general disrepair, cold indoor temperatures, noise, condensation, water leaks from roofs or windows, pest infestations, and rising damp, were also tested in the analysis but were not found to be statistically significant. Local deprivation levels were also tested but were not found to be statistically significant.

**Table 3:** Regression models.

	Probit model (RE): Falling				Probit model (RE): Falling with healthcare use				Heckman (Probit) selection model (RE): Falling						Heckman (Probit) selection model (RE): Falling with healthcare use					
	(1)		(2)		(3)		(4)		(5)		(6)		(7)		(8)		(9)		(10)	
*t-1*	Coeff.	%	Coeff.	%	Coeff.	%	Coeff.	%	Coeff.	%	Coeff.	%	Coeff.	%	Coeff.	%	Coeff.	%	Coeff.	%
Previous fall			0.462***	17.8%			0.337***	13.2%			0.630***	23.6%	0.635***	23.7%			0.370***	14.4%	0.373***	14.5%
Female (ref. male)	0.145***	5.8%	0.107***	4.3%	0.267***	10.5%	0.240***	9.5%	0.132***	5.3%	0.095***	3.8%	0.096***	3.8%	0.243***	9.6%	0.221***	8.7%	0.223***	8.8%
Age	0.025***	1.0%	0.020***	0.8%	0.0243***	1.0%	0.021***	0.8%	0.021***	0.8%	0.021***	0.8%	0.019***	0.8%	0.024***	1.0%	0.024***	1.0%	0.021**	0.8%
Married/Cohabitating (ref. single/widow)	−0.076**	−3.0%	−0.061**	−2.4%	−0.081**	−3.2%	−0.073**	−2.9%	−0.049	−2.0%	−0.0369397	−1.5%	−0.048	−1.9%	−0.077*	−3.1%	−0.075***	−3.0%	−0.089*	−3.5%
Higher education (degree)	0.109***	4.3%	0.089***	3.5%	0.076**	3.0%	0.064*	2.6%	0.106*	4.2%	0.085**	3.4%	0.079**	3.1%	0.060	2.4%	0.046	1.8%	0.040	1.6%
Tenure: Rented housing (private)	0.089	3.5%	0.075	3.0%	0.024	1.0%	0.017	0.7%	0.129*	5.1%	0.103*	4.1%			0.073	2.9%	0.047	1.9%		
Tenure: Rented housing (social)	0.056*	2.2%	0.037	1.5%	0.091**	3.6%	0.082**	3.3%	0.073*	2.9%	0.079**	3.1%			0.106**	4.2%	0.104**	4.1%		
Household size (n)	0.007	0.3%	0.006	0.2%	0.001	0.0%	0.003	0.1%	0.004	0.2%	0.004	0.2%	0.002	0.1%	0.011	0.4%	0.012	0.5%	0.008	0.3%
Number of ADLs	0.125***	5.0%	0.110***	4.4%	0.094***	3.7%	0.075***	3.0%	0.155***	6.2%	0.121***	4.8%	0.123***	4.9%	0.098***	3.9%	0.076***	3.0%	0.079***	3.1%
Number of IADLs	0.102***	4.1%	0.083***	3.3%	0.062***	2.5%	0.049**	2.0%	0.112***	4.5%	0.092***	3.7%	0.089***	3.5%	0.091***	3.6%	0.079**	3.1%	0.073**	2.9%
Fair & Poor health	0.164***	6.5%	0.144***	5.7%	0.163***	6.5%	0.147***	5.8%	0.117***	4.7%	0.106**	4.2%	0.108**	4.3%	0.110**	4.4%	0.101**	4.0%	0.101**	4.0%
Long-term illness/disability	0.140***	5.6%	0.119***	4.7%	0.095***	3.8%	0.075**	3.0%	0.134***	5.3%	0.098***	3.9%	0.100***	4.0%	0.103**	4.1%	0.078**	3.1%	0.081**	3.2%
Electrical/Plumbing issues	0.281***	**11.1%**	0.239**	**9.4%**	0.334***	**13.1%**	0.306***	**12.0%**	0.370***	**14.4%**	0.274**	**10.8%**	0.278***	**10.9%**	0.259**	**10.2%**	0.197*	**7.8%**	0.197*	**7.8%**
Lack of space	0.056*	**2.2%**	0.050*	**2.0%**					0.102**	**4.1%**	0.084*	**3.3%**	0.096**	**3.8%**						
Too dark					0.194**	**7.7%**	0.184**	**7.3%**							0.238**	**9.4%**	0.236**	**9.3%**	0.251***	**9.9%**
Wave 3 (ref. wave = 2)	−0.103**	**−4.1%**	−0.100**	**−4.0%**	**−0.060**	**−2.4%**	**−0.063**	**−2.5%**												
4	−0.171***	−6.8%	−0.155***	−6.2%	−0.192***	−7.6%	−0.186***	−7.4%	−0.165***	−6.6%	−0.178***	−7.1%	−0.177***	−7.0%	−0.1776459	−7.1%	−0.179**	−7.1%	−0.175**	−6.9%
5	−0.122**	−4.9%	−0.086**	−3.4%	−0.169***	−6.7%	−0.149**	−5.9%	−0.091*	−3.6%	−0.079	−3.1%	−0.075	−3.0%	−0.1341191	−5.3%	−0.127*	−5.1%	−0.114*	−4.5%
6	−0.109**	−4.3%	−0.088**	−3.5%	−0.169***	−6.7%	−0.157***	−6.2%	−0.079*	−3.1%	−0.086*	−3.4%	−0.087**	−3.5%	−0.121	−4.8%	−0.122**	−4.9%	−0.120**	−4.8%
7	−0.162***	−6.4%	−0.136***	−5.4%	−0.210***	−8.3%	−0.196***	−7.8%	−0.144***	−5.7%	−0.152***	−6.0%	−0.153***	−6.1%	−0.214	−8.5%	−0.215***	−8.5%	−0.214***	−8.5%
8	−0.124**	−4.9%	−0.089**	−3.5%	−0.156**	−6.2%	−0.137**	−5.4%	−0.097**	−3.9%	−0.083*	−3.3%	−0.084*	−3.3%	−0.085	−3.4%	−0.074	−2.9%	−0.071	−2.8%
9	−0.160***	−6.4%	−0.127***	−5.1%	−0.159**	−6.3%	−0.145**	−5.8%	−0.078	−3.1%	−0.037	−1.5%	−0.059	−2.4%	−0.120	−4.8%	−0.095	−3.8%	−0.135	−5.4%
10	−0.102**	−4.1%	−0.074*	−2.9%	−0.213***	−8.4%	−0.194***	−7.7%												
_cons	−2.605***	−49.5%	−2.300***	−48.9%	−3.404***	−50.0%	−3.233***	−49.9%	−2.184***	−48.6%	−2.305***	−48.9%	−2.198***	−48.6%	−3.277***	−49.9%	−3.349***	−50.0%	−3.136***	−49.9%
N observations	32,596		32,583		32,593		32,580		18,835		18,827		18,837		18,835		18,827		18,837	
N individuals	9,050		9,050		9,050		9,050		6,878		6,877		6,878		6,878		6,877		6,878	
Prob > chi_sq									0.5449		0.3121		0.4503		0.7080		0.5830		0.9125	

Note: p-value: < 0.001 ***; < 0.05 **; < 0.1 *.

Table 3 reports on the different specifications and methods performed. For an easier interpretation, we have also included the effect of housing conditions as percentage points. For instance, reporting plumbing/electrical issues increases the risk of fall by 10.8%. Area-level deprivation (IMD quintiles) was also included in the model as an additional control, but it was not statistically significant and was therefore excluded from the final specification.

In the Heckman selection models, smoking status and number of waves were incorporated as auxiliary variables within the selection equation to strengthen model identification and improve overall model fit. We acknowledge, however, that the appropriateness of smoking status as an exclusion restriction may be questioned, given the established physiological relationship between smoking, frailty, and risk of falls. Consequently, smoking may plausibly exert a direct effect on the outcome beyond its association with sample selection. To examine the robustness of the findings to this assumption, robustness analyses were undertaken excluding smoking status from the selection equation. As shown in Table 4, the results were consistent, indicating that the principal findings were robust to alternative model specifications.

**Table 4:** Robustness analysis.

	Heckman (Probit) selection model (RE): Falling	Heckman (Probit) selection model (RE): Falling with healthcare use
	(1)	(2)
*t-1*	Coeff.	Coeff.
Previous fall	0.622***	0.526***
Female (ref. male)	0.099***	0.234***
Age	0.019***	0.022***
Married/Cohabitating (ref. single/widow)	−0.061**	−0.076**
Higher education (degree)	0.084**	0.058
Tenure: Rented housing (private)	0.090*	0.037
Tenure: Rented housing (social)	0.033	0.049
Household size (n)	0.009	0.004
Number of ADLs	0.115***	0.093***
Number of IADLs	0.115***	0.058**
Fair & Poor health	0.080***	0.131***
Long-term illness/disability	0.097***	0.062**
Electrical/Plumbing issues	0.239**	0.305**
Lack of space	0.070*	
Too dark		0.211**
Wave 3 (ref. wave = 2)	−0.105**	−0.031
4	−0.171***	−0.154**
5	−0.092**	−0.123**
6	−0.080*	−0.118**
7	−0.160***	−0.158**
8	−0.106**	−0.106**
9	−0.101	−0.093
_cons	−2.180***	−3.171***
N observations	29,722	29,717
N individuals	8,584	8,584
Prob > chi_sq (independent eq.)	0.6581	0.6121

Note: p-value: < 0.001 ***; < 0.05 **; < 0.1 *.

### The health and social care costs of falling due to poor housing

Combining branch likelihood with unit costs, we estimated that £1.2 billion annual costs are attributable to falls among older adults living in poor housing. The expected average cost per older people with a fall living in poor housing was £869. Healthcare costs were estimated to be £440 million (£313 per older people), and formal care costs were estimated at £778 million (£556 per older people), both in 2023 prices.

When we compared our estimations to Craig et al.’s work [24], adjusting the Craig figures for inflation and population size, and accounting for the fact that Craig et al. [24] report costs for all older people rather than costs attributable to poor housing, our results were fairly similar in magnitude to the Craig estimate. Adjusted healthcare costs were in our case £1.7 billion (36% of the total) versus £2.4 billion (40% of the total), formal care costs were £2.9 billion (64% of the total) versus £3.5 billion (60% of the total), and a total cost of £4.6 billion versus £5.9 billion. Where differences arose, they were explainable by (i) our focus on a housing-specific subpopulation, and (ii) differences in assumptions about transition probabilities, length of stay, and unit costs. This triangulation provides additional face validity for the modelled results.

We also triangulated our results from a report published by Age UK [45] where health costs of falls were estimated for England, obtaining similar figures: £1.7 billion (versus our £2.0 billion). We couldn’t however obtain the methodology used for this estimation, so we adjust only by inflation and by total cost of falls (rather than confined to those caused by housing conditions). We also attempted to compare our results to a macrosimulation model estimating savings over time associated to fixing home conditions and social care service use [46]. Unfortunately, due to being a project on costs comparison, we were not able to compare findings.

Sensitivity analyses were also conducted to assess the robustness of the cost estimates to key parameter assumptions in the decision tree. While results remained consistent across specifications, total cost estimates were sensitive to parameter variation, ranging approximately from £1 billion to £2 billion.

### Discussion & policy messages

Using information from the English Longitudinal Study of Ageing, we found that around one in three people aged 65 or older experienced at least one fall each year, and approximately one in ten reported having used healthcare services as a result, which is consistent with previous studies [1]. Our analysis also shows that specific housing-related problems, such as electrical or plumbing issues, lack of space, and inadequate lighting, increase the likelihood of falling and of accessing healthcare services following a fall. These effects remained statistically significant across different specifications and modelling approaches. The mechanisms underlying these relationships could plausibly be: water leaks or uneven flooring caused by plumbing problems increase the likelihood of slipping, exposed wires or cluttered cables create tripping hazards, insufficient light makes it harder to identify obstacles, and cramped living spaces restrict movement and contribute to cluttered or obstructed pathways. While other housing-related factors, such as poor insulation or damp, were not statistically significant in our models, this may reflect self-report limitations or strong collinearity across housing problems rather than an absence of effect.

From a financial perspective, we estimated that £440 million and £778 million in annual health and formal care costs, respectively, can be attributed to falls linked to poor housing conditions. The total estimated costs (£1.2 billion) in our study are comparable in magnitude to those reported by Craig et al. (2013) [25] and Age UK (2010) [45] when adjusted for inflation, population, and methodological scope. Moreover, we found that social care costs represent the largest share of the financial burden, highlighting the potential for housing improvements to alleviate pressures on already stretched care systems.

These findings have important implications for policy and practice. Improving housing quality should be recognised as a key component of falls prevention and healthy ageing strategies, alongside clinical and behavioural interventions. The results also highlight the need for stronger collaboration between housing, health, and care sectors to identify individuals at risk and coordinate timely interventions. This aligns closely with the NHS 10-year plan [47], which emphasises the importance of integrated, place-based approaches to improving population health and addressing the wider determinants of wellbeing. Investing in safer, better-quality housing within communities directly supports this agenda, offering a practical route to reduce inequalities and promote healthy ageing at the local level.

Preventing falls through environmental improvements not only represents a health and safety priority but also a means of achieving broader system efficiencies and social benefits. Importantly, many of the improvements needed, such as better lighting, minor repairs, or decluttering and rearranging furniture, are simple and relatively inexpensive to implement, yet can have a significant impact on safety and wellbeing. In this regard, older adults living in poorer-quality housing (often those in rented housing or deprived areas) may be prioritised for housing assessments and adaptation grants. Investment in housing quality can thus serve as an effective form of prevention, reducing hospital admissions and reliance on long-term care services while supporting older people to live independently for longer.

### Limitations

This analysis has several important limitations that should be acknowledged. First, the measure of falls in ELSA is based on self-reports. This may be subject to recall bias or under-reporting due to stigma, memory lapses, or concerns about self-worth, potentially leading to underestimation of fall prevalence. Additionally, ELSA does not provide a breakdown of the type of healthcare used following a fall, so we are unable to distinguish between GP visits, A&E attendances, or walk-in centre consultations. Second, while we took several steps to mitigate potential sources of bias, we recognise that some degree of omitted bias is inevitable in observational research.

Third, the decision tree model used to estimate costs represents a simplified view of the complex pathways through which falls result in healthcare and social care use. While this approach allows for consistent cost estimation, it abstracts from the diversity of real-world trajectories and relies heavily on assumptions and evidence drawn from previous literature. The model also focuses on one-year outcomes and may not capture longer-term consequences of falls. Forth, mental health service use due to anxiety, fear of falling, or other psychological consequences, were not included due to data limitations. Falls can lead to substantial emotional and psychological impacts, potentially resulting in additional service use.

Fifth, the analysis distinguishes between falls associated with poor housing versus other risk factors. However, theoretical differences in these pathways and their downstream consequences have not been fully accounted for. This may contribute to an underestimation of costs. Supportive of this, descriptive analyses in ELSA show that among individuals who experienced a fall, those living in poor housing were slightly more likely to report healthcare use than those in better housing (34.78% vs. 31.15%).

Finally, the accuracy of our cost estimations heavily relies on previous evidence used to populate our decision tree model.

Taken together, these limitations suggest that while our results provide useful estimates of the additional costs of falls associated with poor housing, they are likely to be conservative. Future work may seek to incorporate more detailed information on healthcare use and mental health services, to provide a more comprehensive picture.

## Conclusions

Our findings show that poor housing conditions increase the risk of falls among older people and that associated healthcare and social care costs are significant (estimated at around £1.2 billion annually). Integrating housing improvements as part of falls prevention initiatives could yield important benefits for both individuals and the sustainability of health and care systems.

## References

[1] Office for Health Improvement & Disparities. Guidance - Falls: applying All Our Health. 2022 [accessed on 02.10.2025]. Available online at https://www.gov.uk/government/publications/falls-applying-all-our-health/falls-applying-all-our-health

[2] World Health Organization. WHO global report on falls prevention in older age. 2007 [accessed on 13.02.2025]. Available online at http://www.who.int/ageing/publications/Falls_prevention7March.pdf

[3] Milat AJ, Watson WL, Monger C, Barr M, Giffin M, Reid M. Prevalence, circumstances and consequences of falls among community-dwelling older people: results of the 2009 NSW Falls Prevention Baseline Survey. N S W Public Health Bull. 2011;22(3–4):43–8. doi: 10.1071/NB10065 21631998

[4] Vieira ER, Palmer RC, Chaves PHM. Prevention of falls in older people living in the community. BMJ. 2016;353:i1419. doi: 10.1136/bmj.i1419 27125497

[5] Montero-Odasso M, van der Velde N, Martin FC, Petrovic M, Tan MP, Ryg J, et al. World guidelines for falls prevention and management for older adults: a global initiative. Age Ageing. 2022;51(9):afac205. doi: 10.1093/ageing/afac205 PMC9523684 36178003

[6] NHS England. Going further for winter: Community-based falls response. 2022 [accessed on 14.02.2024]. Available online at https://www.england.nhs.uk/long-read/going-further-for-winter-community-based-falls-response/

[7] Skelton D, Todd C. What are the main risk factors for falls amongst older people and what are the most effective interventions to prevent these falls? World Health Organisation Europe; 2004 [accessed on 13.02.2025]. Available online at https://iris.who.int/bitstream/handle/10665/363812/9789289057318-eng.pdf?isAllowed=y&sequence=1&utm_medium=email&utm_source=transaction

[8] Gale CR, Cooper C, Aihie Sayer A. Prevalence and risk factors for falls in older men and women: The English Longitudinal Study of Ageing. Age Ageing. 2016;45(6):789–94. doi: 10.1093/ageing/afw129 27496938 PMC5105823

[9] Wehner-Hewson N, Watts P, Buscombe R, Bourne N, Hewson D. Racial and Ethnic Differences in Falls Among Older Adults: a Systematic Review and Meta-analysis. J Racial Ethn Health Disparities. 2022;9(6):2427–40. doi: 10.1007/s40615-021-01179-1 34786654 PMC9633486

[10] Bischoff-Ferrari HA, Dawson-Hughes B, Staehelin HB, Orav JE, Stuck AE, Theiler R, et al. Fall prevention with supplemental and active forms of vitamin D: a meta-analysis of randomised controlled trials. BMJ. 2009;339:b3692. doi: 10.1136/bmj.b3692 19797342 PMC2755728

[11] Sherrington C, Michaleff ZA, Fairhall N, Paul SS, Tiedemann A, Whitney J, et al. Exercise to prevent falls in older adults: an updated systematic review and meta-analysis. Br J Sports Med. 2017;51(24):1750–8. doi: 10.1136/bjsports-2016-096547 27707740

[12] Dawson-Hughes B. Effect of vitamin D on risk of falls and fractures - The contribution of recent mega-trials. Metabol Open. 2024;23:100300. doi: 10.1016/j.metop.2024.100300 39100895 PMC11295698

[13] Lewis SR, McGarrigle L, Pritchard MW, Bosco A, Yang Y, Gluchowski A, et al. Population-based interventions for preventing falls and fall-related injuries in older people. Cochrane Database Syst Rev. 2024;1(1):CD013789. doi: 10.1002/14651858.CD013789.pub2 PMC10767771 38180112

[14] Chandola T, Rouxel P. Home modifications and disability outcomes: A longitudinal study of older adults living in England. Lancet Reg Health Eur. 2022;18:100397. doi: 10.1016/j.lanepe.2022.100397 35814336 PMC9257645

[15] Yeoh Lui CX, Yang N, Tang A, Tam WWS. Effectiveness Evaluation of Smart Home Technology in Preventing and Detecting Falls in Community and Residential Care Settings for Older Adults: A Systematic Review and Meta-Analysis. J Am Med Dir Assoc. 2025;26(1):105347. doi: 10.1016/j.jamda.2024.105347 39521020

[16] Cockayne S, Pighills A, Adamson J, Fairhurst C, Crossland S, Drummond A, et al. Home environmental assessments and modification delivered by occupational therapists to reduce falls in people aged 65 years and over: the OTIS RCT. Health Technol Assess. 2021;25(46):1–118. doi: 10.3310/hta25460 34254934 PMC8287374

[17] Clemson L, Mackenzie L, Ballinger C, Close JCT, Cumming RG. Environmental interventions to prevent falls in community-dwelling older people: a meta-analysis of randomized trials. J Aging Health. 2008;20(8):954–71. doi: 10.1177/0898264308324672 18815408

[18] Gillespie LD, Robertson MC, Gillespie WJ, Sherrington C, Gates S, Clemson LM, et al. Interventions for preventing falls in older people living in the community. Cochrane Database Syst Rev. 2012;2012(9):CD007146. doi: 10.1002/14651858.CD007146.pub3 22972103 PMC8095069

[19] Hopewell S, Adedire O, Copsey BJ, Boniface GJ, Sherrington C, Clemson L, et al. Multifactorial and multiple component interventions for preventing falls in older people living in the community. Cochrane Database Syst Rev. 2018;7(7):CD012221. doi: 10.1002/14651858.CD012221.pub2 30035305 PMC6513234

[20] Becker C, Woo J, Todd C. Falls. In: Michel J-P, editor. Jean-Pierre Michel, and others (eds), Oxford Textbook of Geriatric Medicine, 3 edn (Oxford, 2017; online edn, Oxford Academic. Oxford: Oxford Academic; 2017.

[21] Health and Safety Executive. Slips and trips in health and social care: What you need to do. 2024 [accessed on 06.10.2025]. Available online at https://www.hse.gov.uk/healthservices/slips/what-you-need-to-do.htm

[22] Janssen H, Ford K, Gascoyne B, Hill R, Roberts M, Bellis MA, et al. Cold indoor temperatures and their association with health and well-being: a systematic literature review. Public Health. 2023;224:185–94. doi: 10.1016/j.puhe.2023.09.006 37820536

[23] Burns P, Coxon J. Boiler on Prescription Trial: Closing Report. Housing LIN. 2016. Available online at https://www.housinglin.org.uk/_assets/Resources/Housing/Research_evaluation/boiler-on-prescription-closing-report.pdf

[24] Craig J, Murray A, Mitchell S, Clark S, Saunders L, Burleigh L. The high cost to health and social care of managing falls in older adults living in the community in Scotland. Scott Med J. 2013;58(4):198–203. doi: 10.1177/0036933013507848 24215036

[25] Banks J, Batty GD, Breedvelt J, Coughlin K, Crawford R, Marmot M, et al. English Longitudinal Study of Ageing: Waves 0-10, 1998-2023. SN: 5050. 43rd edition. UK Data Service; 2024.

[26] Jones K, Weatherly H, Birch S, Castelli A, Chalkley M, Dargan A, et al. Unit Costs of Health and Social Care 2023 Manual. 2024. doi: 10.22024/UniKent/01.02.105685

[27] NHS England. 2022/23 National Cost Collection Data Publication. 2024. Available online at https://www.england.nhs.uk/publication/2022-23-national-cost-collection-data-publication-2/ [accessed on 13.02.2025].

[28] Turner J, Knowles E, Simpson R, Sampson F, Dixon S, Long J, et al. Impact of NHS 111 Online on the NHS 111 telephone service and urgent care system: a mixed-methods study. Health Services and Delivery Research. Natl Instit Health Care Res. 2021;9(21) [accessed on 10.03.2025]. Available online at https://www.ncbi.nlm.nih.gov/books/NBK575180/pdf/Bookshelf_NBK575180.pdf 34780129

[29] Office for National Statistics. Census 2021. 2021.

[30] Leszczensky L, Wolbring T. How to deal with reverse causality using panel data? Recommendations for researchers based on a simulation study. Sociol Methods Res. 2019;51(2):837–65. doi: 10.1177/0049124119882473

[31] Heckman JJ. Sample selection bias as a specification error. Econometrica. 1979;47(1):153–61. doi: 10.2307/1912352

[32] StataCorp. Stata Statistical Software: Release 18. 2023.https://www.stata.com

[33] Forder J, Fernandez J. Length of stay in care homes. A report commissioned by Bupa. PSSRU Discussion Paper 2769. 2011 [accessed on 14.10.2025]. Available online at https://eprints.lse.ac.uk/33895/1/dp2769.pdf

[34] Hawley S, Inman D, Gregson CL, Whitehouse M, Johansen A, Judge A. Predictors of returning home after hip fracture: a prospective cohort study using the UK National Hip Fracture Database (NHFD). Age Ageing. 2022;51(8):afac131. doi: 10.1093/ageing/afac131 35930719

[35] Png ME, Griffin XL, Costa ML, Achten J, Pinedo-Villanueva R. Utilization and costs of formal and informal care, home adaptations, and physiotherapy among older patients with hip fracture. Bone Joint Res. 2020;9(5):250–7. doi: 10.1302/2046-3758.95.BJR-2019-0221.R1 32566147 PMC7284289

[36] Patel R, Judge A, Johansen A, Marques EMR, Griffin J, Bradshaw M, et al. Multiple hospital organisational factors are associated with adverse patient outcomes post-hip fracture in England and Wales: the REDUCE record-linkage cohort study. Age Ageing. 2022;51(8):afac183. doi: 10.1093/ageing/afac183 36041740 PMC9427326

[37] Pighills AC, Torgerson DJ, Sheldon TA, Drummond AE, Bland JM. Environmental assessment and modification to prevent falls in older people. J Am Geriatr Soc. 2011;59(1):26–33. doi: 10.1111/j.1532-5415.2010.03221.x 21226674

[38] Mason S, Knowles E, Colwell B, Dixon S, Wardrope J, Gorringe R, et al. Effectiveness of paramedic practitioners in attending 999 calls from elderly people in the community: cluster randomised controlled trial. BMJ. 2007;335(7626):919. doi: 10.1136/bmj.39343.649097.55 17916813 PMC2048868

[39] Mackenzie L, McIntyre A. How Do General Practitioners (GPs) Engage in Falls Prevention With Older People? A Pilot Survey of GPs in NHS England Suggests a Gap in Routine Practice to Address Falls Prevention. Front Public Health. 2019;7:32. doi: 10.3389/fpubh.2019.00032 30915322 PMC6421941

[40] Lavedán A, Viladrosa M, Jürschik P, Botigué T, Nuín C, Masot O, et al. Correction: Fear of falling in community-dwelling older adults: A cause of falls, a consequence, or both? PLoS One. 2018;13(5):e0197792. doi: 10.1371/journal.pone.0197792 29771978 PMC5957385

[41] Age UK. Intermediate care and reablement. Factsheet 76. 2025 [accessed on 14.10.2025]. Available online at https://www.ageuk.org.uk/siteassets/documents/factsheets/fs76_intermediate_care_and_reablement_fcs.pdf

[42] Beresford B, Mayhew E, Duarte A, Faria R, Weatherly H, Mann R, et al. Outcomes of reablement and their measurement: findings from an evaluation of English reablement services. Health Soc Care Community. 2019;27(6):1438–50. doi: 10.1111/hsc.12814 31368621 PMC6851672

[43] NHS. Care after illness or hospital discharge (reablement). 2025 [accessed in 14.10.2025]. Available online at https://www.nhs.uk/social-care-and-support/care-after-a-hospital-stay/care-after-illness-or-hospital-discharge-reablement/

[44] Age UK. Celebrating 100 successful Strength & Balance courses. 2024 [accessed on 14.10.2025]. Available online at https://www.ageuk.org.uk/northtyneside/about-us/news/articles/2024/celebrating-100-successful-strength-balance-courses/

[45] Age UK. Falls in the over 65s cost NHS £4.6 million a day. 2010 [accessed on 09.10.2025]. Available online at https://www.ageuk.org.uk/latest-press/archive/falls-over-65s-cost-nhs/

[46] Hu B, Brimblecombe N, Cartagena-Farias J, Silva-Ribeiro W. Projected costs of long-term care for older people in England: The impacts of housing quality improvements. Health Policy. 2025;152:105246. doi: 10.1016/j.healthpol.2025.105246 39799693

[47] Department of Health and Social Care. NHS 10 Year Health Plan for England: fit for the future. Policy paper. 2025 [accessed on 14.10.2025]. Available online at https://www.gov.uk/government/publications/10-year-health-plan-for-england-fit-for-the-future

